# RNA Interference Is Enhanced by Knockdown of Double-Stranded RNases in the Yellow Fever Mosquito *Aedes aegypti*

**DOI:** 10.3390/insects11060327

**Published:** 2020-05-27

**Authors:** David Giesbrecht, Daniel Heschuk, Ian Wiens, David Boguski, Parker LaChance, Steve Whyard

**Affiliations:** 1 Department of Biological Sciences, University of Manitoba, Winnipeg, MB R3T 2N2, Canada; d.gies@ymail.com (D.G.); heschukd@myumanitoba.ca (D.H.); wiensi3@myumanitoba.ca (I.W.); parker.lachance@gmail.com (P.L.); 2 Department of Fisheries and Oceans, Winnipeg, MB R3T 2N2, Canada; daveboguski@gmail.com

**Keywords:** nuclease, RNAi, mosquito, *Aedes*, dsRNase

## Abstract

RNA interference (RNAi) techniques are being developed for a range of pest insect control technologies, including the sterile insect technique (SIT) and double-stranded RNA (dsRNA)-based insecticides. In SIT applications, where >99% of the released males should be sterile to meet industry standards, the efficiency of RNAi will need to be improved for many insect species if this technology is to be adopted. Endogenous dsRNases can impede dsRNA delivery in some insects, and, here, we investigated whether dsRNases in the midgut could limit RNAi efficacy in the mosquito *Aedes aegypti*. Ten putative dsRNases were identified in the *Ae. aegypti* genome, with two highly expressed in the midguts of larvae. Using an ex vivo assay, we observed that dsRNA was rapidly degraded within the mosquito larva’s gut. Double-stranded RNA targeting these two dsRNases, when fed to the larvae, effectively reduced gut dsRNase activity. When these dsRNase-specific dsRNAs were co-delivered with dsRNA targeting a cyan fluorescent protein (CFP) reporter gene, greater knockdown of CFP fluorescence was observed. These results suggest that inhibiting dsRNase activity could enable the implementation of RNAi-based mosquito control methods.

## 1. Introduction

RNA interference (RNAi) is a well-established reverse genetics method used to explore gene functions in insects. While being an indispensable research tool in many insect species (reviewed in [1,2,3]), RNAi responses are highly variable, with some insects having only limited RNAi functionality [1,4,5]. The variability in RNAi responses in different insects has been attributed to a broad range of factors, including variations in the double-stranded RNA (dsRNA) stability within different insects, differences in cellular uptake of the dsRNA trigger molecules, differences in intracellular distribution and processing of the dsRNAs, and differences in systemic distribution of the effector molecules [6,7]. Of these factors, the most extensively studied is the stability of the dsRNAs when delivered to the insects. RNAi-mediated knockdown of gene transcripts is achievable in many insect species by injecting dsRNA directly into the hemocoel, but as this delivery method can be technically challenging and/or time-consuming, many researchers have sought to develop feeding formulations to administer dsRNA for higher throughput applications. While many successful dsRNA feeding experiments have been reported [6,8], a growing number of research groups have observed that dsRNases within the insects can greatly reduce the efficacy of RNAi [1,7].

In some insects, gut dsRNases can degrade the ingested dsRNA within minutes, thereby preventing RNAi, despite the insect having the essential core RNAi machinery. In locusts, for example, direct injection of dsRNA into the hemocoel was used to knock down target transcripts, but ingested dsRNAs were rapidly degraded by gut dsRNases, thereby preventing effective RNAi [9]. In the same study, the authors found that knockdown of two dsRNases improved the RNAi response in the Colorado potato beetle *Leptinotarsa decemlineata* [9]. In the locusts, however, knockdown of four dsRNases proved ineffective at restoring the RNAi response, which the authors suggested was either due to incomplete knockdown of all four dsRNases, or other unidentified dsRNases were still active. In the pea aphid *Acyrthosiphon pisum,* dsRNases have similarly been shown to reduce RNAi efficiency [10]. Chung and colleagues [11], working with the same species, reported that an RNAi response was only observed when a dsRNase was knocked down together with the genes they were investigating. RNAi in lepidopteran species is generally poor, often only achieving modest knockdown of transcripts if high doses of dsRNA are used. Like other insects, dsRNases have been found to contribute to low RNAi efficiency in the silkworm moth *Bombyx mori* [12] and in the Oriental corn borer *Astrinia furnicalis* [13]. DsRNases have also been implicated in reducing RNAi efficiency in a dipteran pest, the Queensland fruit fly *Bactrocera tryoni*. Co-delivery of dsRNAs targeting two *B. tryoni* dsRNases and one target gene dramatically improved knockdown of the target transcripts within three days of feeding on the dsRNA mixture [14].

In mosquitoes, many researchers choose to deliver dsRNA by direct hemocoel injections, to ensure precise dsRNA doses and consistent knockdown of the genes under study [15,16,17,18,19]. DsRNA feeding in mosquitoes has proven effective in inducing RNAi, but, typically, only partial reductions in the target transcripts or limited knockdown phenotypes have been described [20,21,22]. Improvements in RNAi efficacy in mosquitoes have been observed when the dsRNA was fed to larvae either encapsulated [23,24] or expressed within microorganisms [25,26,27,28]. In these latter cases, the enhanced RNAi efficacy was attributed to protection of the ingested dsRNA from gut dsRNases.

Gut dsRNases in mosquitoes and their possible impacts on RNAi efficacy have not been described before. In this study, we used phylogenetic analyses to identify a diversity of nucleases in the mosquito *Aedes aegypti*. Two nucleases expressed in the larval gut were suspected of contributing to variable RNAi responses in this insect. Using bacterially produced short (21 to 26 nucleotide (nt) hairpin dsRNAs (shRNAs), in a manner similar to that described by Hapairai and colleagues [9,10,13,14], we examined whether RNAi efficiency can be enhanced by knocking down the activity of two of these dsRNases, thereby enabling other dsRNAs to reduce their target transcripts more effectively.

## 2. Materials and Methods

### 2.1. DsRNase Expression Profiling and Phylogenetic Analyses

Ten *Ae. aegypti* genes were identified by homology with *Drosophila melanogaster* dsRNAse gene CG6839 and to dsRNases in several agricultural pest insects, including *Ostrinia furnicalis* [13], *Acyrthosiphon pisum* [10], *Leptinotarsa decemlineata* [9], and *Bactrocera tryoni* [14]. Mosquito homologs to AAEL008858 were identified from current Vectorbase gene sets, and a neighbor-joining tree was created in MEGA X: Molecular Evolutionary Genetics Analysis across computing platforms. Primers for each *Ae. aegypti* gene were designed using NCBI Primer-BLAST (Basic Local Alignment Search Tool). Wild-type mosquitoes used in this study were Liverpool strain *Ae. aegypti* obtained through BEI Resources, NIAID (National Institute of Allergy and Infectious Diseases, National Institutes of Health *Aedes aegypti*, strain LVP-IB12, eggs, MRA-735, contributed by David W. Severson. This colony was maintained at 12:12 light:dark photoperiod at 28°C with heparinized rat blood provided weekly and 10% sucrose provided *ad libitum*. Eggs were collected on paper towel and hatched into ddH_2_O bubbled with nitrogen to induce rapid hatching. Larvae were dissected immediately post-hatch (0 h) and at 6 time points until at 48 h. Isolated guts and remaining carcasses were dissected from pools of 10 mosquitoes, and RNA was extracted from the pooled tissues, or from individual intact insects, using the GeneJet RNA extraction kit (Thermo Fisher Scientific, Waltham, MA, USA) according to manufacturer’s specifications. RNA was treated with DNase-I (Thermo). The complementary DNA (cDNA) was prepared by reverse transcription with qScript (Quantabio, Beverly, MA, USA) in 10 uL reactions, and 20 uL qPCR reactions were performed with SsoFast Evagreen supermix (BioRad, Hercules, CA, USA) using the following thermocycling conditions: Three min at 94 °C, 40 cycles of 94 °C for 20s and 57 °C for 20s, followed by melt-curve analysis on a CFX Connect PCR machine (BioRad). Transcript abundance relative to the ribosomal S7 gene (primer sequences are found in Table S1) was calculated using the Livak method [29] for gut and carcass and L4 and adult females. Melt-curve analyses confirmed that only single amplicons were produced for all primer sets. As the PCR efficiencies for all primers ranged between 96 and 102% (Table S2), the single reference gene was considered appropriate for all transcript-level comparisons. Relative transcript abundances were calculated and expressed using a heat map generated in Microsoft Excel.

### 2.2. Knockdown of DsRNases by Feeding ShRNAs Expressed in Bacteria

To reduce the activity of dsRNases in vivo, mosquito larvae were fed bacteria expressing shRNAs, targeting either a dsRNase gene or enhanced cyan fluorescent protein (eCFP). For treatments where two genes were targeted, mosquitoes were fed a mixture of two *Escherichia coli* strains. Target sequences were identified using Integrated DNA Technology’s online dicer substrate short interfering RNA (siRNA) design tool, and short hairpin RNAs (shRNAs) were designed with a stem sequence of 21–26 base pair (bp) and a loop sequence of 9 bp (Figure S1, Table S3). Annealed DNA oligos of the shRNA sequences were blunt-end ligated into the expression vector pJET1.2 (Thermo Fisher Scientific) downstream of a T7 promoter. A single shRNA construct was expressed by each *E. coli* strain. A non-specific control shRNA was synthesized containing a *Discosoma* Red (DsRed) sequence. *E. coli* (HT115 (DE3), available from University of Minnesota Caenorhabditis Genetics Center) cells were transformed with the plasmids, grown to mid-log phase (~16 h), and expression was induced with 0.4 M isopropyl β-d-1-thiogalactopyranoside (IPTG) for 4 h before harvesting. Harvested cells (40 mL) were pelleted by centrifugation at 5000 × g for 5 min and the pellets were then suspended in 4 mL 1% agar supplemented with 1 mL 10% sterile brewer’s yeast (MP Biomedicals) slurry. This mixture solidified in open-topped 5-mL syringes and sliced into 0.5-mL feeding pellets. Mosquito eggs were hatched synchronously by submerging them in deoxygenated, previously boiled ddH_2_O into which nitrogen gas was bubbled for 2 min. In 100-mm petri dishes, 40 larvae in 20 mL ddH20 were provided with two pellets. These pellets were the exclusive food of the larvae from 4 h post hatch until the fifth day of development, when they were dissected to measure impact on dsRNase activity.

### 2.3. Ex Vivo Degradation Assays

Midguts were dissected in phosphate buffered saline (PBS) from bacterially fed fourth instar larvae, taking care to eliminate Malpighian tubules, crop, and gut contents sheathed within the peritrophic membrane. To standardize damage to the gut tissues, all guts were torn with forceps at five places. Pools of three guts were incubated overnight at 4 °C in 100 uL PBS before use in ex vivo degradation assays. To measure dsRNA degradation, 100 ng of dsRNA specific for the bacterial galactosidase (*Gus)* gene [25] was incubated with 7 uL of mosquito gut secretions in 1 × PBS pH 7.4 (Thermo Fisher Scientific). Aliquots of each sample were incubated at 28 °C for 0, 10, 30, or 60 min. Enzyme activity was halted by transferring the samples to ice. For negative controls, gut extracts were either heat-killed (20 min at 80 °C) before incubating with dsRNA for 60 min or contained only PBS with no gut extracts added. Samples for each of 4–8 biological replicates, with 2–4 technical replicates, were loaded onto 1.5% agarose gels and resolved for 30 min at 120 V in 1×Tris-acetate buffer (TAE) buffer (Thermo Fisher Scientific). Gels were stained with ethidium bromide and imaged under UV illumination using a Gel Doc XR+ system (BioRad). The Band Analysis tools of Image Lab software, version 4.1, (BioRad) were used to calculate band intensities relative to heat-killed controls. For each time point, relative band intensity of RNAi treatments was compared to DsRed controls using an unpaired Wilcoxon rank sum test.

### 2.4. Co-Feeding of DsRNase and Reporter Gene ShRNA

To determine whether knockdown of dsRNases by shRNA improves RNAi when co-delivered with other RNAi triggers, we targeted the eCFP gene in the *Aedes aegypti* Orlando Gr3[eCFP] strain obtained from BEI Resources (Atlanta, GA) [30]. The eCFP gene in this mosquito strain was expressed under the control of the ubiquitin promoter, and, hence, the fluorescent protein was expressed in all tissues throughout development, allowing for measurement of impact both in the gut and in the carcass. For co-feeding experiments 26 nt hRNAs expressed in *E. coli* were used with bacterial feeding pellets provided as the exclusive food from the day of hatching until fluorescent protein quantification. More stringent expression parameters were used compared to 22mer expression used for degradation assays. An overnight starter broth was grown to mid-log phase and 1 mL of this broth was used to inoculate 40 mL of LB broth. This culture was grown to an optical density (OD) of 0.4 and induced with 0.4 mM Isopropyl β- d-1-thiogalactopyranoside (IPTG), then grown to an OD of 1.0. Bacteria expressing a scramble nt shRNA were used as controls. Bacteria from 40 mL of each dsRNase-gene shRNA expression strain was mixed 1:1 with double-stranded RNA against eCFP (dseCFP) bacteria. An additional 2 ×dseCFP control treatment was produced with cells from 80 mL of culture broth, and feeding pellets were made as described above. Twenty mosquitoes were reared in 5 mL of water in deep-well, 100-mm petri dishes. Following development on bacterial feeding pellets, fluorescence was measured in whole bodies of L4 mosquitoes, 5 days after hatching. RNAi efficiency against a fluorescent reporter was quantified using methods modified from previous studies in *Caenorhabditis elegans* [31]. To minimize size effects of background fluorescence, only larvae of uniform size were selected, and this size selection produced varying numbers of larvae analyzed for each treatment. CFP fluorescence intensity was measured by placing a single live larva in 5 ul 1:1 1 × PBS:pure glycerol (to reduce their mobility) in each well of concave-bottomed 96-well plates and read using a Biotek Synergy H1 plate reader (excitation 435 nm, emission 505 nm).For these readings, a 7 × 7 grid with 600 nm spacing captured the entire bottom of each well, and the mean of the highest 10 of 49 values (indicative of the larva’s position within the well) was expressed as a percentage of fluorescence relative to background autofluorescence levels observed in non-CFP mosquitoes treated in the same manner. The mean of each treatment was compared with a Wilcoxon sum-rank test. Survival of mosquitoes treated with 8858 combined with eCFP shRNA treated was calculated by observing movement of larvae for a 30-s period on day five. Survival was expressed as a proportion alive of a starting number of 20 larvae per petri dish. This was replicated four times for scramble nt controls and 8858 combined with eCFP shRNA. Adjusted mortality was calculated using Abbott’s formula and the means of survival per dish were compared with a Wilcoxon rank-sum test.

### 2.5. Measurement of DsRNA Uptake and Degradation in the Mosquito Hemolymph

To quantify dsRNA in the hemolymph of mosquito larvae following soaking with long dsRNA, fourth instar *Ae. aegypti* larvae were soaked in 10 uL of 0.1 ug/uL ~350 bp Gus dsRNA [25]. Glass microcapillaries were used to withdraw approximately 0.5 uL of hemolymph from each larva. RNA from hemolymph of 10 pooled individuals was extracted and quantified relative to ribosomal S7 as described above.

## 3. Results

### 3.1. Multiple DsRNases Are Found in Mosquitoes

**Figure 1:**
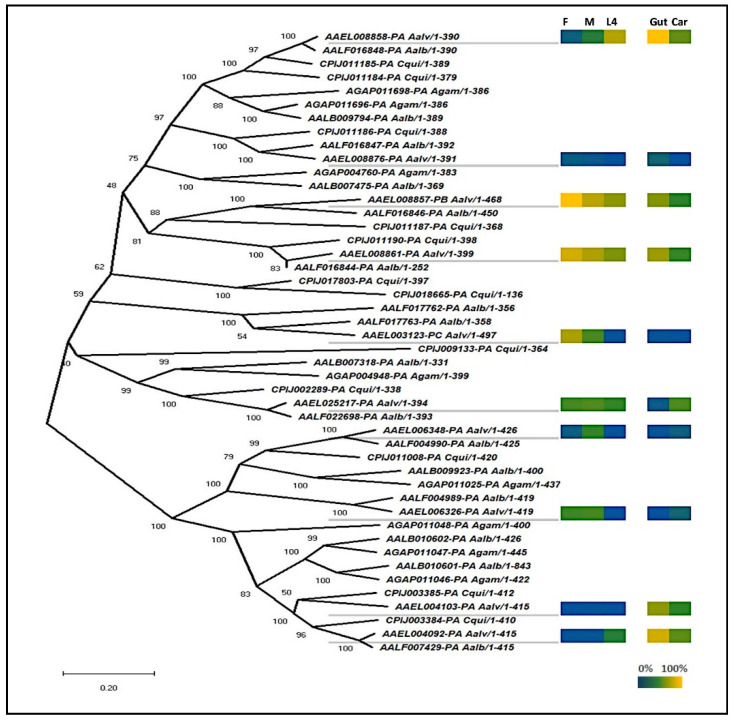
Phylogenetic tree of mosquito dsRNases and expression heat map of representative *Ae. aegypti* dsRNases. The neighbor-joining phylogenetic tree was produced in MEGA X from Clustal-Omega alignments’ mosquito homologue proteins with scale bar indicating genetic distance. F, M, and L4 represent male, female, and fourth instar larvae, respectively. Gut and Car represent gut and carcass. Expression is shown by a color scale where 100% is equal to the highest observed expression and 0% is the limit of detection from four biological replicates per sample type.

Sequences similar to other insect dsRNases [9,10,13,14] were identified using BLAST searches of the publicly available mosquito datasets (Vectorbase gene set AaegL5.2). Phylogenetic analyses revealed that aedine mosquitoes have a diversity of dsRNases, with the greatest number found in *Ae. aegypti* (n = 10) and *Ae. albopictus* (n = 10) (Figure 1). The 10 *Ae. aegypti* genes had a maximum E-value of 3e-27 at the amino acid level to the *D. melanogaster* gene CG6839 annotated a dsRNase. In addition, all 10 proteins were predicted by ProSite to include the same functional domains as previously described dsRNases found in other insects [10,13], with a signal peptide and a dsRNA cleavage domain. In all but one (AAEL006326) of the *Ae. aegypti* genes, the dsRNA cleavage domain included a conserved histidine residue that was functionally shown to be a proton acceptor in the well-described *Serratia marcescens* dsRNase homologue [32] (Figure S2). In addition, most the *Ae. aegypti* dsRNase genes are found clustered on three regions on chromosome 3 (Table S4). 

### 3.2. Two DsRNases Are Expressed in the Gut of Larval Ae. Aegypti

**Figure 2:**
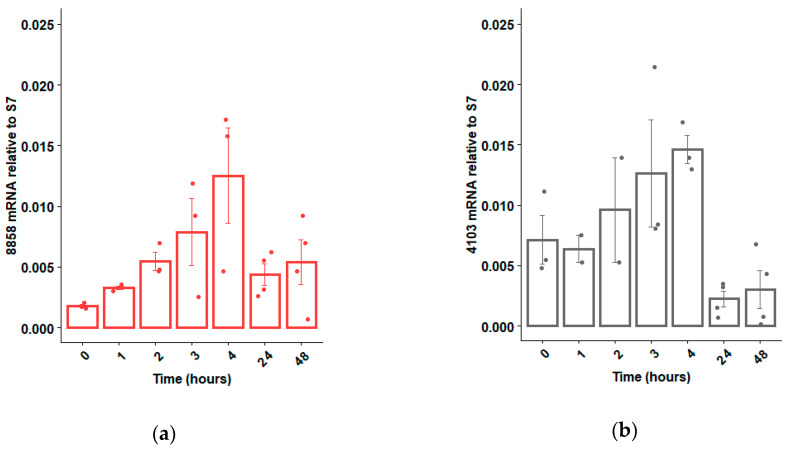
Expression profile of dsRNase genes 8858 (**a**) and 4103 (**b**) from pools of 10 *Ae. aegypti* larvae immediately post-hatch (0 h) to 48 h (early fourth instar). Expression was determined by qRT-PCR using ribosomal S7 as a control reference gene. Individual biological replicates (ranging between n = 2 and n = 4) and standard error are shown by dots and error bars, respectively.

If RNAi technology is to be used to sex-sort or sterilize *Ae. aegypti* in a sterile insect technique (SIT) program, an ideal time to deliver the dsRNA would be during the larval feeding period, when dsRNA can be administered cheaply and efficiently to many insects simultaneously [33]. Hence, we opted to focus our attention on dsRNases expressed within the guts of larvae, as these dsRNases would be the first to contact the administered dsRNAs. Based on qRT-PCR analyses, six of the 10 putative dsRNases were expressed mostly in the guts, relative to the rest of the body, and two of those dsRNase genes were more strongly expressed in larvae relative to adults (Figure 1). These two larval gut-specific genes, AAEL008858 and AAEL004103 (henceforward described as 8858 and 4103), were expressed throughout larval development, except during the initial hours post-hatching, when very low transcription was observed for 8858 (Figure 2a,b). Expression of 8858 increased in some individuals between 3 and 4 h post-hatch (Figure 2b), corresponding to the inflation of the head capsule and initiation of feeding [34]. Thereafter, expression of 8858 was variable.

### 3.3. Knockdown of DsRNases Reduces DsRNA Degradation in the Gut

**Figure 3:**
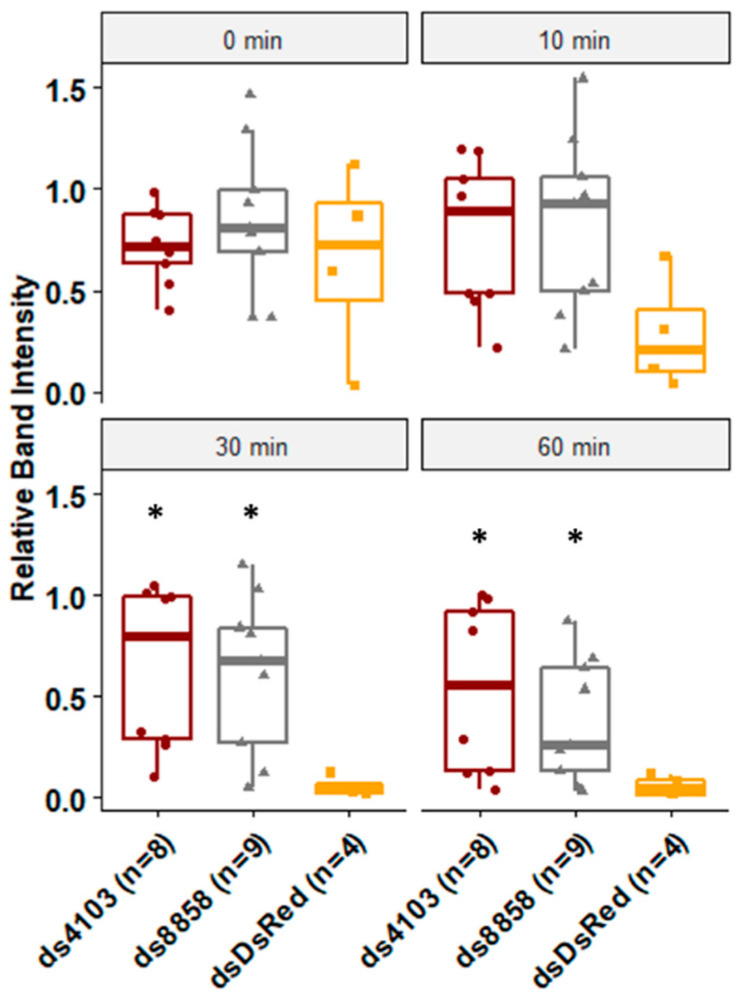
Degradation of long dsRNA after exposure to gut-derived enzymes. The shRNA were expressed in *E. coli* and fed to *Ae. aegypti* larvae in brewer’s yeast-supplemented agar feeding pellets for five days at 28°C. Midguts were dissected and soaked in PBS overnight. The resulting enzyme mix was co-incubated with long dsRNA for 0, 10, 30, or 60 min and resolved by gel electrophoresis. Band intensity was calculated relative to heat-killed controls. Biological replicates are shown as jittered points (number of replicates ranged from four to eight per treatment). Wilcoxon rank-sum *p*-values less than 0.05 relative to DsRed controls are indicated by *, comparisons are for both ds4103 and ds8858 to dsDsRed for each time point.

To assess whether the two dsRNases had a role in dsRNA degradation in the gut, larvae were fed *E. coli* expressing 22nt shRNAs targeting either 8858 or 4103. After five days of continuous feeding, degradation of dsRNA by midgut secretions was assessed in ex vivo assays. No dsRNA degradation was detected in dsRNA samples lacking gut extracts or in samples treated with heat-treated gut extracts. Gut extracts from negative control larvae that were fed bacteria expressing non-specific double-stranded RNA against DsRed (dsDsRed) degraded virtually all dsRNA within 30 min, indicating the presence of highly potent dsRNase activity in the gut tissues. In contrast, larvae fed ds4103 showed no significant degradation of dsRNA for the first 60 min. Larvae fed ds8858 showed no significant degradation of dsRNA for the first 30 min, although by 60 min, most of the dsRNA was subsequently degraded (Figure 3).

### 3.4. Co-Delivery of DsRNase ShRNA with a Target ShRNA Enhances RNAi Efficiency

**Figure 4:**
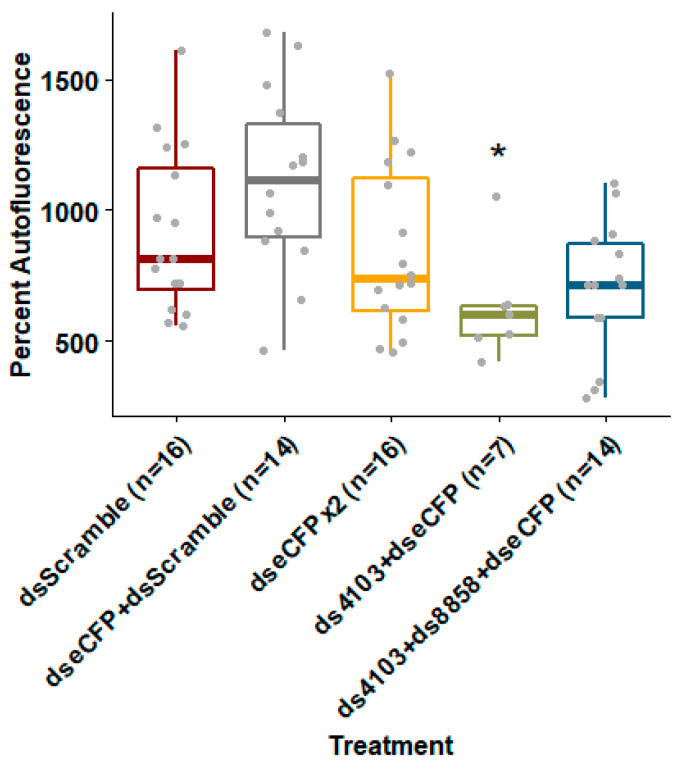
Co-feeding eCFP shRNA with dsRNase shRNA improved depletion of fluorescent protein by RNAi. The shRNA were expressed in *E. coli* and fed to *Ae. aegypti* larvae in brewer’s yeast-supplemented agar feeding pellets for five days at 28 °C. Whole larvae of uniform size were placed in conical-bottom 96-well plates and eCFP fluorescence was measured using a Biotek Synergy H1 plate reader. Box-plots show mean and upper and lower quartiles with range whiskers. Wilcoxon rank-sum *p*-value less than 0.05 relative to dsScramble controls are indicated by *.

To test whether co-feeding shRNAs against 8858 and 4103 could improve knockdown of a fluorescent transgene’s expression, we co-delivered *E. coli* expressing shRNA against dsRNase genes and eCFP to an *Ae. aegypti* strain expressing eCFP under control of a ubiquitin promoter. Larvae fed bacteria expressing shRNAs targeting eCFP alone or together with a negative control “scrambled nt” shRNA failed to reduce eCFP fluorescence. Doubling the concentration of bacteria expressing eCFP shRNA resulted in only an 8% (*p* = 0.127) reduction in fluorescence compared to scrambled shRNA controls. However, when larvae were co-fed bacteria expressing shRNA targeting both eCFP and 4103, eCFP fluorescence was reduced by 32% (*p* = 0.018) relative to the negative shRNA controls (Figure 4). Co-feeding all three shRNAs (eCFP, 4103, and 8858) resulted in a non-significant reduction in fluorescence of 24% (*p* = 0.09). Treatments with the 26 nt shRNA targeting 8858 and eCFP suffered from adjusted mortality of 56% (n = 4, Wilcoxon *p* = 0.029) compared to “scrambled nt” shRNA controls (data shown in Table S5). No significant mortality was observed in any of the other treatments. Variation in number of larvae analyzed per treatment was due to size selection prior to fluorescence measurements.

### 3.5. DsRNA Enters the Hemolymph within Minutes of DsRNA Soaking

**Figure 5:**
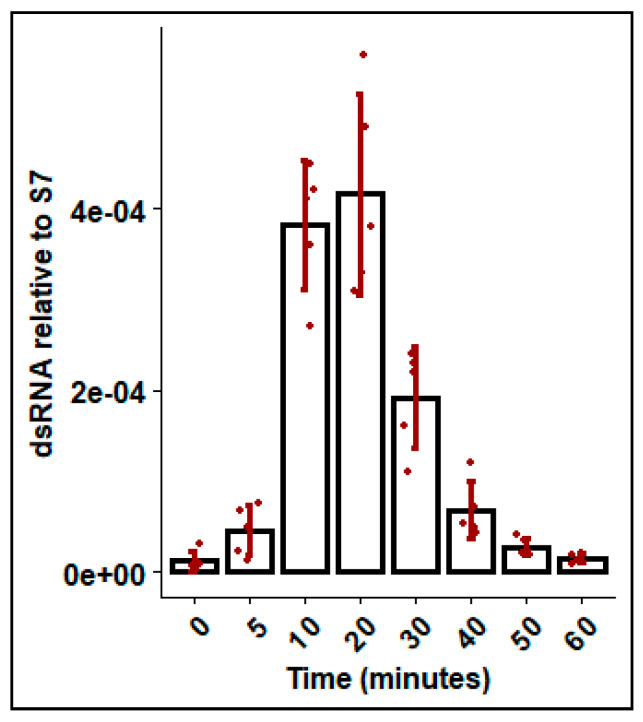
DsRNA recovered in hemolymph following soaking in dsGus for durations between 0 and 60 minutes. Fourth instar *Ae. aegypti* larvae were soaked in ~350 bp dsGus dsRNA 0.1 ng/uL and hemolymph was removed by microcapillary and dsRNA was measured by qRT-PCR, with ribosomal S7 used as a reference gene. Error bars represent standard deviations and bars represent mean of biological replicates (n = 4), shown as jittered points.

To explore dsRNA uptake by mosquitoes from the aquatic environment, mosquitoes were soaked in *Gus* dsRNA and the dsRNA present in the hemolymph was measured at various time points. DsRNA was detected in the hemolymph within the first 5 min of exposure, and levels steadily increased for the first 20 min. Thereafter, levels declined rapidly and dsRNA was not detectable at 60 min post exposure (Figure 5).

## 4. Discussion

RNAi efficiency in insects is variable and the factors contributing to this variability are still not well understood [7]. DsRNases have been shown to reduce RNAi efficiency by degrading RNAi trigger molecules in the gut, preventing uptake in target tissues [35]. The impact of dsRNases on dsRNA degradation and reduced RNAi efficiency in mosquitoes was previously unknown. Here, we identified several putative dsRNases in the mosquito *Ae. aegypti*, and focused our attention on two dsRNases expressed in the larval gut. Knockdown of one these dsRNases, 4103, proved effective in improving RNAi in the larval mosquitoes.

Functional predictions and phylogenetic analyses suggest that dsRNases, (Figure 1) with possible tissue and developmental specialization, are abundant in mosquitoes. From the publicly available data, aedine mosquitoes have the greatest diversity of dsRNases, with 10 predicted in *Ae. aegypti* and 10 in *Ae. albopictus*. In *Ae. aegypti*, the 10 dsRNases were found in close proximity on chromosomes 2 and 3, which suggests that the proliferation of these genes may have arisen through gene-duplication events. For example, genes AAEL008857, AAEL008858, AAEL008861, and AAEL008876 were found on a region of chromosome 3 spanning 24.3 kb (Table S4) and share highly similar amino acid sequences. Both of these aedine mosquitoes are relatively indiscriminate in their selection of freshwater breeding habitats, can develop in diverse aquatic environments, and outcompete local species in both man-made and naturally occurring aquatic habitats [36]. We speculated that these dsRNase genes may have proliferated to permit exploitation of diverse habitats and food sources in both adult and larval stages. Viral and eukaryotic dsRNA is encountered in the larval and adult diet, and dsRNases would undoubtedly assist in the degradation of these nucleic acids within the gut lumen. Interactions between viral pathogens and their mosquito hosts typically begin within the gut [37], and dsRNases in this tissue may provide a first line of defense by degrading viral dsRNA within the gut lumen. A variety of pathogens are known to use dsRNAs to facilitate the infection process, and, hence, degrading these nucleic acids within the gut could protect the insect host. Virally encoded double-stranded microRNAs (miRNAs), for example, have been implicated in modulating host gene expression, and, more recently, eukaryotic protozoan parasites have similarly been observed to modulate immune responses in insect hosts [37,38]. Aside from offering possible protection from pathogens, dsRNases may also enable mosquito larvae to maximize nutrient acquisition from ingested microorganisms, as free nucleotides are a source of bioavailable phosphate in nutrient-poor aquatic habitats [39]. The increased mortality rates of the larvae feeding on 8858 shRNA may indeed be a consequence of the larvae failing to digest free nucleotides in the environment.

Our results suggest that dsRNA in the gut is degraded primarily by secreted dsRNases. Both *Ae. aegypti* RNase 4103 and 8858 were found to be necessary for dsRNA degradation ex vivo, as shown by shRNA knockdown. While it is possible that other enzymes may be contributing to dsRNA degradation in the larval gut, the improved durability of the dsRNA following the knockdown of these two dsRNases suggests that other dsRNases have only a small contribution to dsRNA degradation in the gut lumen. Previous reports of dsRNA degradation in *Ae. aegypti* assessed both processing of dsRNA by the RNAi machinery and degradation by dsRNases, suggesting that processing is a key limiting factor in dsRNA feeding experiments. [35]. Poor efficiency of dsRNA processing may be a factor limiting the success of RNAi experiments in *Ae. aegypti*, but our results suggest that a phenotypic response can be achieved by reducing dsRNase activity. Taken together, our results and other reports of a high degree of gene knockdown by dsRNA feeding [26] suggest that dsRNA processing in *Ae. aegypti* is sufficient to induce a strong RNAi response.

Based on our observation that dsRNAs were found within the hemolymph within minutes of ingestion, the durability of dsRNA in the early stages of feeding will be critical to improving the efficacy of the dsRNA at mediating knockdown in mosquito larvae, particularly if the dsRNAs must move systemically to reach targets beyond the gut. Co-feeding larvae with dsRNase-specific dsRNAs along with eCFP dsRNA proved effective at improving knockdown of that gene’s proteins over several days. While the first ingestion of either ds1403 or ds8858 would not have immediate impact on their respective dsRNase levels in the gut, their activity was clearly reduced after five days, leading to improved knockdown of the reporter gene. Curiously, the dsRNA levels in the hemolymph decreased after 20 min, suggesting that further transfer of the dsRNA to the hemolymph had ceased. At this point in our research, it is not clear whether the insects had stopped feeding after this time point or if some mechanism of dsRNA sequestration was occurring. Future experiments are planned to assess whether dsRNA transfer rates are slowing after 20 min or if other dsRNases in the mosquitoes may be contributing to the rapid disappearance of dsRNA in the hemocoel.

The variation in RNAi efficiency in many insects may be the consequence of dsRNases rapidly degrading exogenously applied dsRNA. We speculated that a small, consistent dose of dsRNA is sufficient to induce RNAi, but in many insects, dsRNA was degraded before entering gut epithelial cells. Steady delivery of dsRNA [26] or daily dosing [25,27] in some insects can ensure that gene knockdown is initiated and sustained. However, for some insects, only highly concentrated doses of dsRNA were effective at knocking down targeted genes [4]. In many of these cases, despite most of the dsRNAs being depleted by dsRNase degradation, enough persisted to knock down the targeted genes. At lower dsRNA concentrations, or with only sporadic feedings of dsRNA, an insufficient amount of dsRNA may survive dsRNase degradation within the gut to target the transcripts at the peak of their expression. Delivery of naked dsRNA also leaves the dsRNA vulnerable to degradation, whereas encapsulating the dsRNA can protect it from dsRNases. In previous studies where *Ae. aegypti* were soaked in siRNAs, RNAi-induced phenotypes were observed in less than 50% of individuals but approached 100% when larvae were continuously fed bacteria or yeast expressing shRNA [26]. A combination of reduction in dsRNase activity, as reported here and in other insects [10,14], combined with protection inside the cells of a microorganism expression system [25,40] or liposomes [14], may be an effective approach for many species. Such an approach may provide the sustained delivery of dsRNA needed for consistent transcript depletion and application of RNAi in pest management.

Successful induction of RNAi by feeding dsRNA is challenging, but overcoming these challenges will yield rewards to pest control. The possibility of co-delivery of dsRNase-specific dsRNA with their genes of interest may enable low-cost reverse genetics using RNAi, even against gene targets previously dismissed as unresponsive. This may include genes essential for male fertility and female-specific splice variants of sex determination genes. A promising extension of the current work is the use of RNAi enhanced by dsRNase knockdown to improve reliability of RNAi-based SIT. Coupled to low-cost dsRNA production systems in microorganisms, such a system may bring large scale SIT by RNAi within reach.

## 5. Conclusions

Mosquito guts were found to have enzymes that degrade dsRNA. By knocking down two of these enzymes using RNAi, we were able to reduce dsRNA degradation and improve RNAi-mediated knockdown of a fluorescent reporter. Our next steps will be to co-deliver dsRNA targeting transcripts of genes required for both gut dsRNases and sperm development, with the goal of achieving more reliable sterility by RNAi.

## Supplementary Materials

The following are available online at https://www.mdpi.com/2075-4450/11/6/327/s1, Figure S1: construct schematic of shRNA expression vectors in pJET1.2 cloning vector for all shRNAs used in this study, Figure S2: Clustal Omega alignment of 10 *Ae. aegypti* putative dsRNase genes aligned to *Serratia marcescens* nucA (P13717). The predicted proton acceptor histidine residue is highlighted in yellow, Table S1: Primer sequences used in qRT-PCR assays in this study, Table S2: Primer efficiency data for qRT-PCR assays used in this study, Table S3: shRNA constructs used in this study, Table S4: Locations of ten *Ae. aegypti* dsRNase genes and current VectorBase (VB) community annotation status. Approximate locations of these genes on chromosome map are shown with arrows, R^2^ values and percent efficiency (E%) are shown for each primer set (Table S2) and tissue type. PCR reactions with insufficient data are shown as not determined (ND), Table S5: Mortality observed in ds8858 26 nt shRNA feeding experiments compared to dsScramble controls. Survival was scored after five days of feeding on shRNA expressing bacteria in petri dishes with 20 larvae per dish. Abbott’s adjusted mortality for all ds8858+dseCFP = 56.3% (n = 80).
