# Supplementary material for: RNA Interference Is Enhanced by Knockdown of Double-Stranded RNases in the Yellow Fever Mosquito Aedes aegypti
Source: Insects. 2020 May 27;11(6):327. doi: 10.3390/insects11060327 (PMC7349537; doi:10.3390/insects11060327)
Supplement: Supplementary file 1 [file insects-11-00327-s001.pdf]

insects

MDPI

Supplementary Materials

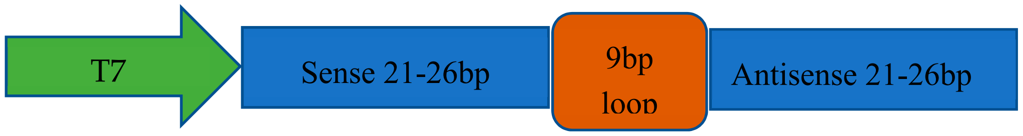

Figure S1. construct schematic of shRNA expression vectors in pJET1.2 cloning vector for all shRNAs used in this study.

```
sp|P13717|NUCA_SERMA         --------PALNP------ADTLAPADYTGANAALKVDRGHQAPLASLAGVSDWESLNYL 129
AAEL004103-PA                TNYLYTVNKQRQTIAQILQSQDLADDIVRDVNSGIYMARGHIAARVDFIYGTQQNATFWF 259
AAEL004092-PA                INTLYTVNRQRQTLATVLGSQAIADDLVRDASTGIFMARGHIAARADFIYGTQQNATFWF 259
AAEL006348-PA                MTKLYNIESQRKTFEKILGSSARADALLN-SKQDMFLARGHLAAKADFVFGAHQRATFWF 271
AAEL006326-PA                MAEIYSFDVQHATLGLILGSTNRANNLLN-RRKDIFIAKGRLAAQADFVYGSQQAATFRY 264
AAEL003123-PC                VSSAYNQESQLNRLVALFGADPNPWGSAEVYYNLSYLQRGHLVPDADQLFTTWQWSTYFY 346
AAEL025217-PA                LSTVYTQNSQRTRLANLLGSEELA--KQYISSS-SFFAKGHLTPDGDAVLNSWAGATYFY 245
AAEL008857-PB                ADALYTQTTQFERFEALLGSKAQA--EKYIEPGKTFLNRGHLTPRGDGIFQTWKHATFFY 290
AAEL008861-PA                PSTSYTQASQLERLTTLLGSAEQA--SRFVFTN-SFMARGHMSPDADGIYRSWQFTTYFF 246
AAEL008876-PA                PATSYTQQAQLTRLTELLGSEEQA--KKFISGGSYYMARGHLAPDADGIYRSWQWATFFY 241
AAEL008858-PA                PASSYTTASQATRLAVLLGSQAQA--DRFITTS-SYMSRGHLSPDADGIFRSWQWATYFY 240
                                                :                . :*:     .    :   :
```

Figure S2. Clustal Omega alignment of 10 *Ae. aegypti* putative dsRNase genes aligned to *Serratia marcescens nucA* (P13717). The predicted proton acceptor histidine residue is highlighted in yellow.

Table S1 Primer sequences used in qRT-PCR assays in this study.

Accession #	GeneID	5′-3′ forward primer sequence	5′-3′ reverse primer sequence
**AAEL009496**	*S7rp*	AAATAAATTCGCTATGGTTTTC	CCTTCTTGCTGTTGAACTCG
**AAEL004092**		CGAGTGGAATAAACTAGCTTCAG	CAAGCTCCAAGTATTCGCCT
**AAEL002042**		ATGGTGACGCTGTGTTGAAC	GAGGGTCAGAACGTCGTAGA
**AAEL004103**	4103	AGATTCGCTCCATCCTGCTC	TGAACCCATTGCTGCAAACG
**AAEL006348**		AGAACCGCTGTAGAATCGCA	GCCCAAAGAAATTTTACAGCCGT
**AAEL006326**		GTACAACGGGGACTTCGACA	GCTCTACCTGTTCACCGCTT
**AAEL003123**		AGCATACGTGGAATAAATCTGGT	TTGGACTCGAGCTGCATCTG
**AAEL008876**		GGAGCTCACCTGGGAAGAAC	GACGCTGTATTGTTGTTTGCAT
**AAEL008858**	8858	CCGTTTTGTTGGGTTCGCAA	GCGTTGACAACCTGCCATTG
**AAEL008857**		GATTCGACTCCAAGCTGCTC	ACACCGATGCACTGGGATAC
**AAEL008861**		GGACGCGGATGGAATCTACC	GTTGACAACCTGCCACTTCG

Table S2. Primer efficiency data for qRT-PCR assays used in this study. R^2 values and percent efficiency (E%) are shown for each primer set (Supplementary Table 2) and tissue type. PCR reactions with insufficient data are shown as not determined (ND).

Gene	Female gut		Female carcass		Larva gut		Larva carcass	
	**R^2**	**E%**	**R^2**	E%	R^2	E%	R^2	E%
**AAEL009496**	0.995	97.9	0.992	101.1	0.991	97.2	0.989	102.1
**AAEL002042**	0.977	101.6	0.986	99.2	0.982	97.8	0.972	96.1
**AAEL003123**	0.995	101.7	ND	ND	0.998	100.9	ND	ND
**AAEL004092**	0.989	98.5	ND	ND	0.991	99.1	ND	ND
**AAEL004103**	0.991	98.5	0.987	99.9	0.996	101.1	0.984	101.8
**AAEL006326**	0.988	96.2	ND	ND	0.984	97.8	ND	ND
**AAEL006348**	0.99	98.9	0.989	96.3	0.997	96.7	ND	ND
**AAEL008857**	0.986	95.9	0.982	96.4	0.986	96.9	0.982	95.8
**AAEL008858**	0.994	96.8	0.991	97.4	0.992	96.9	0.989	97.8
**AAEL008861**	0.949	97.6	ND	ND	0.988	99.2	ND	ND
**AAEL008876**	0.987	96.8	0.982	96.2	0.991	98.8	0.995	97.4

Table S3. shRNA constructs used in this study.

Strain Number	*E. coli* strain	Gene target and length	Target Sequence 5′-3′
1	HT115 (DE3)	pJET-8858 22mer	ACAAGTACCTCTTGCGGTGGCG
2	HT115 (DE3)	pJET-4103 22mer	ATTGCTCGGAGCTAGGAGTGAA
3	HT115 (DE3)	pJET-8858 26mer	CCTGTTCTGGAATTCTGGCGAGACTA
4	HT115 (DE3)	pJET-4103 26mer	ATATTGCTCGGAGCTAGGAGTGAATC
5	HT115 (DE3)	pJET-eCFP 21mer	CCATGATATAGACGTTGTGGCTG
6	HT115 (DE3)	pJET-scramble 23mer	GTATAGTATAGTATACCGTATAA
7	HT115 (DE3)	pJET-DsRed 23mer	ACCGTGAAGCTGAAGGTGACCAA

Table S4. Locations of ten *Ae. aegypti* dsRNase genes and current VectorBase (VB) community annotation status. Approximate locations of these genes on chromosome map are shown with arrows.

Gene	Genome Location	Description
**AAEL025217**	Chromosome 2: 203659053	No description
**AAEL008861**	Chromosome 3: 236574296	deoxyribodsRNAse I, putative [Source:VB Community Annotation]
**AAEL008857**	Chromosome 3: 236610049	deoxyribodsRNAse I, putative [Source:VB Community Annotation]
**AAEL008876**	Chromosome 3: 236632143	deoxyribodsRNAse I, putative [Source:VB Community Annotation]
**AAEL008858**	Chromosome 3: 236643493	deoxyribodsRNAse I, putative [Source:VB Community Annotation]

**AAEL003123**	Chromosome 3: 277923934	deoxyribodsRNAse I, putative [Source:VB Community Annotation]
**AAEL006348**	Chromosome 3: 396923473	deoxyribodsRNAse I, putative [Source:VB Community Annotation]
**AAEL006326**	Chromosome 3: 396927710	deoxyribodsRNAse I, putative [Source:VB Community Annotation]
**AAEL004103**	Chromosome 3: 401525211	deoxyribodsRNAse I, putative [Source:VB Community Annotation]
**AAEL004092**	Chromosome 3: 401536506	deoxyribodsRNAse I, putative [Source:VB Community Annotation]

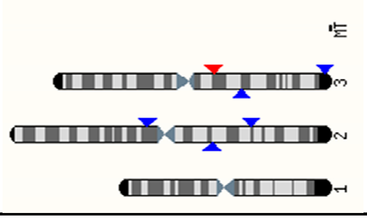

Table S5. Mortality observed in ds8858 26mer shRNA feeding experiments compared to dsScramble controls. Survival was scored after five days of feeding on shRNA expressing bacteria in petri dishes with 20 larvae per dish. Abbott's adjusted mortality for all ds8858+dseCFP = 56.3% (n = 80).

Treatment	Replicates	Mean Survival	Standard Error
**dsScramble**	4 (n=80)	0.88	0.037
**ds8858+dseCFP**	4 (n=80)	0.38	0.062
**ds4103+dseCFP**	2 (n=40	0.83	0.053
**2x dseCFP**	2 (n=40)	0.90	0.071
**ds4103+ds8858+dseCFP**	2 (n=40)	0.70	0.035

© 2020 by the authors. Licensee MDPI, Basel, Switzerland. This article is an open access article distributed under the terms and conditions of the Creative Commons Attribution (CC BY) license (http://creativecommons.org/licenses/by/4.0/).
